# Clinicopathological significance of *c-KIT* mutation in gastrointestinal stromal tumors: a systematic review and meta-analysis

**DOI:** 10.1038/srep13718

**Published:** 2015-09-09

**Authors:** Lin Yan, Lei Zou, Wenhua Zhao, Yansen Wang, Bo Liu, Hongliang Yao, Haihua Yu

**Affiliations:** 1 Department of oncology, Shandong Jiaotong Hospital, Jinan, 250031, P.R.China; 2 Department of gastrointestinal surgery, Shandong Cancer Hospital, Jinan, 250017, P.R.China; 3 Department of oncology, Shandong Provincial Qianfoshan Hospital, Shandong University, Jinan, 250014, P.R.China; 4 Department of gastrointestinal surgery, Shandong Provincial Qianfoshan Hospital, Shandong University, Jinan, 250014, P.R.China; 5 Department of General Surgery, Second Xiangya Hospital of Central South University, Changsha 410011, China

## Abstract

Many types of *KIT* mutations have been observed in gastrointestinal stromal tumors (GISTs), but their prognostic and predictive significance are still unclear. A meta-analysis and literature review were conducted to estimate the contribution of *KIT* mutations in prognostic parameters and clinic-pathological significance of GISTs. A total of 18 relevant articles from PubMed, EMBASE and Web of Science databases were included in this study. The frequency of *KIT* mutation was significantly increased in the GIST patients with higher mitosis (≥5/50 high-power fields (HPFs) and larger size (≥5 cm) of tumors than in those with lower MI (≤5/50HPFs) and smaller size (≤5 cm) of GISTs respectively. The rate of *KIT* mutation was not significantly changed between GISTs in stomachs and in small intestines. *KIT* mutational status has prognostic significance for patients’ outcome. GIST patients with *KIT* exon 9 mutations have higher risk of progression than those with exon 11 mutations. 5 year relapse-free survival (RFS) rate was significantly higher in patients with *KIT* exon 11 deletion than in those with other type of *KIT* exon 11 mutations. The deletion involving *KIT* exon 11, particularly codons 557–558, is a valuable predictor of prognosis for patients with GISTs.

Gastrointestinal stromal tumors (GISTs) are the most common mesenchymal tumors in the gastrointestinal (GI) tract. The population-based studies showed that the annual incidence of GISTs ranges from 11 to 19.6 per million population^1^,^2^. It has been a considerable debate regarding their cellular origin and diagnosis^3^. After gain-of-function mutations in the c-*KIT* protooncogene were discovered in 1998, GISTs were reliably distinguished from other histopathological subtypes of GI mesenchymal tumors^4^. GISTs occur primarily in older patients of either sex^5^, which are present anywhere along the GI tract from esophagus to the rectum, most commonly located in stomach (60%) and small intestine (25%) 5. Local recurrence and metastasis are frequently observed in patients with GISTs after adequate resection and adjuvant therapy with tyrosine kinase inhibitor (TKIs)^6^. In addition, metastasis to the lung and bones occurs in patients with advanced disease^6^. Therefore, it is critical to identify risk criteria to predict their recurrence and metastasis.

c-*KIT*, the cellular homologue of the oncogene v-*KIT*, was isolated from feline fibrosarcoma, the Hardy-Zuckerman 4 feline sarcoma virus (HZ4-FeSV). The viral genome of HZ4-FeSV contains a new oncogene that was designated v-*KIT*, which encodes a transmembrane tyrosine kinase receptor called KIT^7^. Huizinga *et al.* revealed that mice with mutations in the *KIT* gene lacked the network of interstitial cells of Cajal which was associated with Auerbach’s nerve plexus and intestinal pacemaker activity, indicating that the interstitial cells of Cajal express the KIT receptor^8^. Mutations of the *KIT* gene in GISTs occur most frequently in *KIT* exon 11 (juxtamembrane domain), followed by *KIT* exon 9 (extracellular domain), less frequently, mutations occur in the adenosine triphosphate (ATP)-binding pocket (exon 13) or activation loop (exon 17) (Fig. 1)^5^,^9^. Many types of *KIT* mutations have been observed in GISTs, but controversy still exists concerning their prognostic and predictive value^10^. Deletions in the *KIT* exon 11 most frequently involve the 5′ portion between codons 550 and 560^11^. A few studies have shown that tumors containing deletion in this area are clinically more aggressive than tumors with other type of mutations. However, several studies have reported inconsistent results^6^,^12^,^13^,^14^. The aim of this study is to estimate the contribution of different types of *KIT* mutations in prognostic parameters and clinic-pathological significance of GISTs.

## Methods

### Search strategy and selection criteria

**Figure 2:**
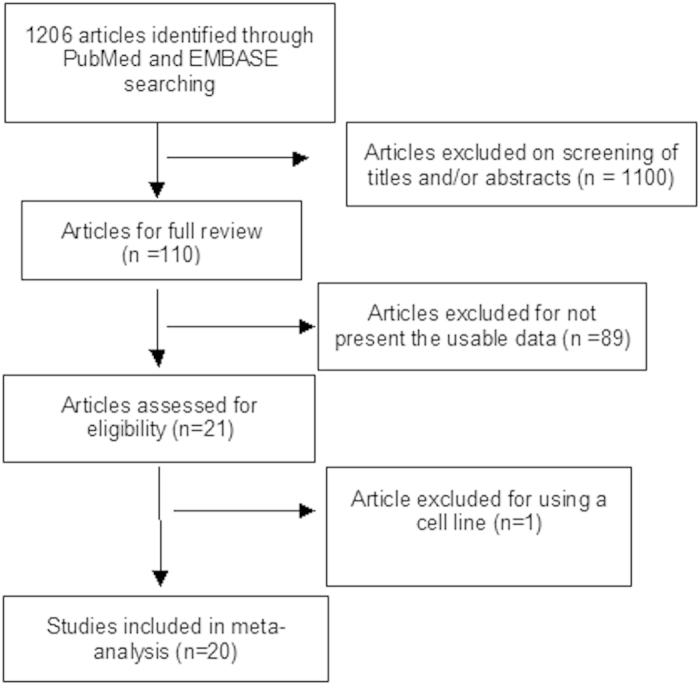
Schematic flow diagram for selection of included studies.

We conducted comprehensive literature searches in the PubMed, EMBASE and Web of Science databases in September 2014 with no low limit set for date of publication, using the following keywords: c-*KIT* or KIT and GIST or gastrointestinal stromal tumor. The language was limited to English and Chinese. A total of 1206 articles were identified with the initial search. Inclusion criteria for study selection were: 1) The articles in which the association between c-*KIT* mutation and the clinicopathological significance of GIST was evaluated; 2) The articles in that the association between c-*KIT* mutations and prognosis in patients with GIST was evaluated. Exclusion criteria were: 1) The studies which used the same population or overlapping database; 2) The studies of *in vitro* cell culture models; 3) The studies which showed insufficient data to calculate Odds Ratio or Hazard Ratio (Fig. 2). The search identified 18 articles of which were eligible for quantitative analysis in this meta-analysis. The detailed information of 18 relevant citations is listed in Table 1.

### Data extraction and study assessment

Two investigators (LY and LZ) independently extracted data and reviewed the contents of the articles to determine whether or not they met the criteria for inclusion. Any discontent was discussed and resolved by a consensus including other two investigators (WZ and YW). A data extract form was developed accordingly. One review author (KL) extracted the following data from the included studies: first author’s name, year of publication, number of patients, mitosis number per 50 HPFs in GISTs, size of GISTs, and c-*KIT* mutation status. The second author (LX) checked the extracted data, and disagreement was resolved by the discussion with other two authors (BL and HY) for all issues.

### Statistics analysis

All analysis was performed with Review Manager 5.2. Heterogeneity between studies was assessed using the Q-test and *I*^2^ index. Odds Ratio (OR) with 95% confidence intervals were calculated by using a fixed or random effect model depending on heterogeneity (a fixed effect model for *I*^2 ^≤ 50%, a random effect model for *I*^2 ^> 50%). Meta-analysis was performed to compare 5 year relapse free survival (RFS) in c-*KIT* exon 11 deletion and other type of c-*KIT* mutations in patients with GIST. C-*KIT* mutation frequency was compared in different size and different MI of tumors. The multivariate HRs were collected, and the log HRs and its standard errors were calculated for individual study. Pooled hazard ratio (HR) with a 95% confidence interval was calculated for the association between the risk of GISTs and c-*KIT* mutation status. All p values were two sided. Funnel plots were used for detection of publication bias. A sensitivity analysis, in which one study was removed at a time, was conducted to assess the result stability.

## Results

Flow chart for study selection is reported in Fig. 2. There were 18 relevant articles available for meta-analysis, which included 3938 patients. The following items were collected from each study: first author’s name, year of publication, number of patients, countries, the number of mitosis per 50 HPFs in GIST, tumor size, c-*KIT* mutation, treatment and the time of follow-up (Table 1).

**Figure 3:**
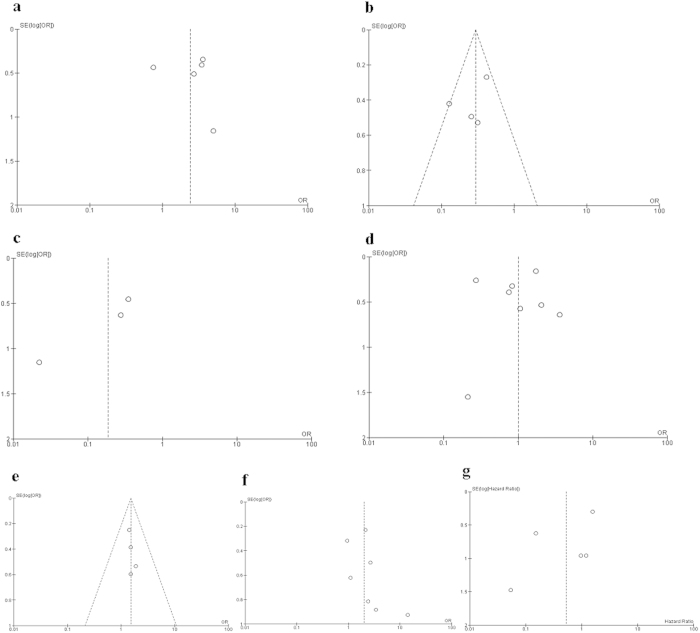
Funnel plot for publication bias. (**a**) Forest plot for PFS of GIST patients with *KIT* exon 11 mutation and *KIT* exon 9 mutation; (**b**) 5 year RFS of GIST patients with *KIT* 11 exon deletion and other *KIT* 11 exon mutation; (**c**) 5 year RFS of GIST patients with codons 557–558 of *KIT* 11 exon deletion and other *KIT* 11 deletion d : *KIT* mutation of patients with GIST in stomach and small intestine; (**d**) *KIT* mutation in different size of GIST; (**f**) *KIT* mutation in different of mitosis index of GIST; (**g**) the association of c-*KIT* mutation and the risk of GIST.

The quality of each study was assessed with the Newcastle Ottawa Quality Assessment Scale (NOQAS). These scales were utilized to allocate a maximum of nine points for the quality of selection, comparability, exposure, and outcomes for study participants. Of the studies, one scored 8 points, ten scored 7 points, six scored 6 points, and one scored 5 points. Hence, the studies were of a relatively high quality (data not shown). The funnel plots were largely symmetric (Fig. 3) suggesting there were no publication biases in the meta-analysis of c-*KIT* mutation and clinicopathological features. We conducted a sensitivity analysis by removing a single study at one time. The pooled HR was not significantly changed, indicating the stability of our analyses.

**Figure 4:**
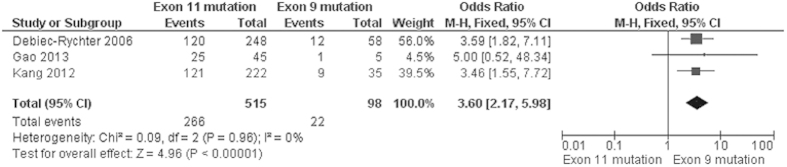
Forest plot for PFS of GIST patients with *KIT* exon 11 mutation and *KIT* exon 9 mutation.

**Figure 5:**
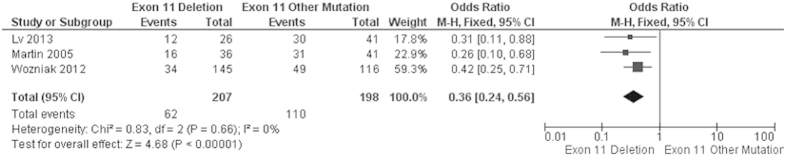
Forest plot for 5 year RFS of GIST patients with *KIT* 11 exon deletion and other *KIT* 11 exon mutations.

**Figure 6:**
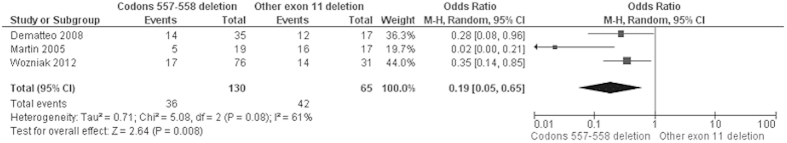
Forest plot for 5 year RFS of GIST patients with Codons 557–558 of *KIT* 11 exon deletion and other *KIT* 11 exon deletions.

**Figure 7:**
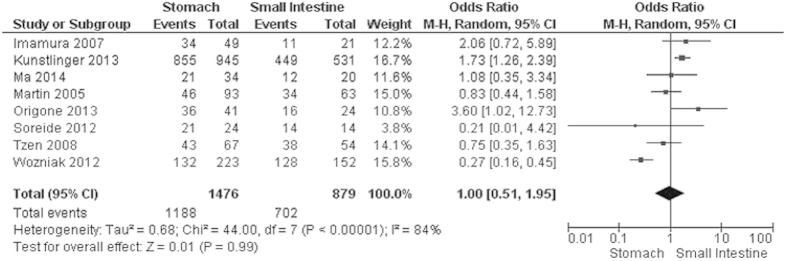
Forest plot for *KIT* mutation of patients with GIST in stomach and small Intestine.

**Figure 8:**
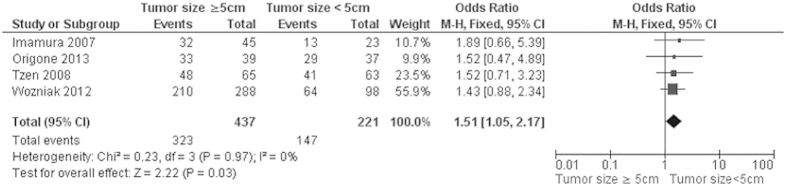
Forest plot for *KIT* mutation in different size of GIST.

**Figure 9:**
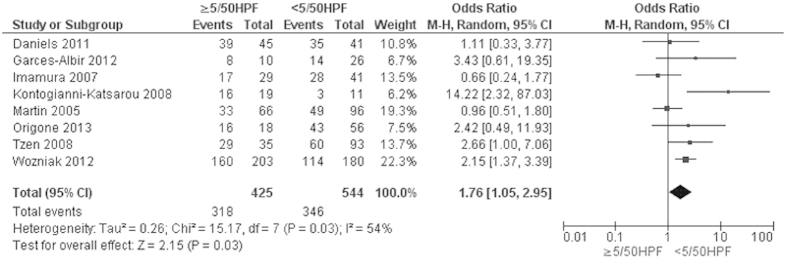
Forest plot for *KIT* mutation in different of mitosis index of GIST.

**Figure 10:**
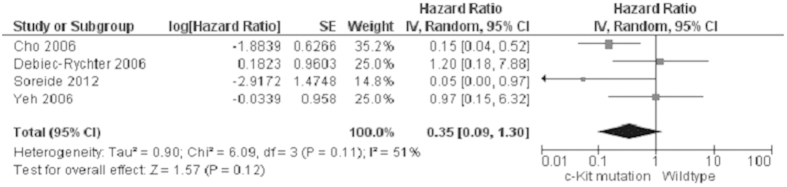
Forest plot for the association of *KIT* mutation and the risk of GIST. Checklist S1. A PRIMA checklist.

Progression-free survival (PFS) of GIST patients was significantly worse in patients with *KIT* exon 9 mutations than in those with *KIT* exon 11 mutations, OR was 3.60, 95% CI 2.17–5.98, z = 4.96, p < 0.00001, heterogeneity *I*^2 ^= 0% (Fig. 4). 5-year RFS rate was significantly lower in patients with *KIT* exon 11 deletion than in those with other type of *KIT* exon 11 mutations, OR was 0.36, 95% CI 0.24–0.56, z = 4.68, p < 0.00001, heterogeneity *I*^2 ^= 0% (Fig. 5). Moreover, RFS for 5 year was significantly worse in patients with GISTs bearing deletions involving *KIT* codon 557–558 than in those bearing other deletions of *KIT* exon 11 (Fig. 6). The rate of *KIT* mutation was not significantly changed between GISTs in stomachs and those in small intestines, OR was 1.00, 95% CI 0.51–1.95, z = 0.01, p = 0.99, heterogeneity *I*^2 ^= 84%, p < 0.00001 (Fig. 7). *KIT* mutations were significantly more frequently observed in the patients with larger size (≥5 cm) of GISTs than those with smaller size (≤5 cm) of GISTs, OR was 1.51, 95% CI 1.05–2.17, z = 2.22, p = 0.03, heterogeneity *I*^2 ^= 0%, p = 0.97 (Fig. 8). *KIT* mutation was significantly increased in the patients with higher mitosis index (MI) (≥5/50 HPFs) of GISTs compared to the patients with lower MI (≤5/50 HPFs) of tumors. OR was 1.76, 95% CI 1.05–2.95, z = 2.15, p = 0.03, heterogeneity *I*^2 ^= 57%, p = 0.03 (Fig. 9). *KIT* mutations were not significantly associated with the risk of mortality of patients with GIST. Hazard Ratio (HR) was 0.35 with a 95% confidence interval (CI) 0.09–1.30, z = 1.57, p = 0.12, heterogeneity *I*^2^ = 51% (Fig. 10).

## Discussion

GISTs are the tumors with KIT expression, located in the gastrointestinal tract. Gain of function mutations in either KIT or platelet-derived growth factor receptor alpha (PDGFRA) were found in about 80%–85% of case^4^,^15^,^16^. Many types of *KIT* mutations involved in exon 9, 11, 13 and 17 have been described in GISTs, including point mutation, insertion, deletion and duplication^5^. Treatment with tyrosine kinase inhibitor (TKIs) is effective in reducing disease recurrence after primary surgery and controlling unresectable disease^17^. Therefore, it is essential to identify mutation status to predict its response to TKIs and prognosis. Our analysis showed that *KIT* mutation was not associated with the risk of mortality of patients with GISTs. In the future, the stratified analysis by tumor size and mitosis index should be carried out to identify the prognosis power of *KIT* mutation, because tumor size and mitosis index are the most important confounding factors. In addition, the overall survival of patients with GISTs may depend on the specific type of *KIT* mutation. We performed a detailed subgroup analysis of relationship between different types of *KIT* mutations and prognosis of patients with GISTs. The result indicated that PFS of GIST patients was significantly worse in *KIT* exon 9 mutations than in *KIT* exon 11 mutations. Previous studies indicated the response to imatinib treatment was worse in patients whose tumors harbored *KIT* exon 9 mutations than in those with *KIT* exon 11 mutations^18^,^19^. Patients with GIST treated with imatinib in all three studies were included in present meta-analysis (Fig. 2). There was no bias created from different treatments. Thus, GIST patients with *KIT* exon 9 mutations have higher risk of progression than those with exon 11 mutations.

Interestingly, deletions in the *KIT* exon 11 most frequently involve the 5′ portion between codons 550 and 560, and less frequently involve codons 562–579^12^,^13^,^20^. There is no significant difference in the response rate of imatinib or median progression-free survival among the patients with exon 11 deletion, point mutations and mixed-type mutations^21^,^22^. Our result showed 5-year RFS was significantly worse in patients with *KIT* exon 11 deletion than in those with other type of *KIT* exon 11 mutations. Moreover, RFS for 5 year was significantly worse in codon 557–558 deletion of *KIT* exon 11 than other deletion of *KIT* exon 11. Recently, a few studies reported controversial results of RFS for five year in patients of GIST with codon 557–558 deletion and other deletion of *KIT* exon 11 due to the small size of patient samples^12^,^13^,^14^,^20^. For the first time, we pooled four studies in this meta-analysis with a total of 127 patients and more precisely assessed RFS for five year in patients of GIST with different parts of *KIT* exon 11 deletion.

*KIT* is a member of type III receptor tyrosine kinase family that contains platelet-derived growth factor receptors-α and -β (PDGFRA and PDGFRB), as well as the macrophage colony stimulating- factor receptor (CSF1R) and the Fl cytokine receptor (FLT3)^23^. Mutations of the *KIT* gene in GISTs occur most frequently in *KIT* exon 11, the juxtamembrane domain that disrupts the normal juxtamemberane secondary structure and activate downstream signaling pathways, including the MAP kinase pathway (RAF, MEK, and ERK), the PI3 kinase/AKT pathway, and STAT3^24^,^25^,^26^. The MAP and PI3 kinase pathway upregulate important transcriptional factors and lead to cell proliferation, and they downregulate the cell cycle inhibitor p27^KIP^ as well as anti-apoptotic signaling. Therefore, *KIT* mutation is a potential predictive factor for prognostic implication. We compared the frequency of *KIT* mutations in different size of tumors and different MIs. Our result indicated that *KIT* mutation was significantly more frequent in the patients with larger size ≥5 cm) and higher MI (≥5/50 HPFs) of GIST than in patients with smaller size (≤5 cm) and lower MI (≤5/50 HPFs) of GIST respectively. Taniguchi *et al.* have reported that there is a direct relationship between the presence of mutation in tumor size and mitotic count^27^, which is in agreement with our result. Previous studies revealed that tumors larger than 5 cm and the presence of more than 5 mitoses/50 HPF were clearly associated with worse outcome^28^. Tumor size and mitotic counts traditionally have been the two factors for estimation of prognosis^29^. Zhao *et al.* conducted a meta-analysis and found that incidence of MI (>5/50 HPFs) is not significantly higher in patients with mutated *KIT* than in the patients with wild type *KIT*^30^. This discrepancy could be due to relatively small sample size (1751 patients). Present meta-analysis included 3980 patients and the result is more accurate. Taken together, our study indicated that *KIT* mutation status is another evaluable factor to estimate prognosis in GISTs in addition to tumor size and mitotic counts.

*KIT* exon 11 deletion may be associated with the risk of mortality of patients with GISTs. Additional research in the future especially larger prospective studies will be needed to evaluate this relationship. Finally, our study only selected the published articles, but it did not include some relevant unpublished papers which may result in certain publication bias. Thus the result should be interpreted carefully.

In conclusion, *KIT* mutational status has prognostic significance for patients with GISTs. GIST patients with *KIT* exon 9 mutations have higher risk of progression than those with exon 11 mutations. The deletion of *KIT* exon 11, particularly codon 557–558 deletion of *KIT* exon 11, was a valuable predictor of prognosis for patients with GISTs. The frequency of *KIT* mutation was significantly increased in the GIST patients with higher mitosis (≥5/50 HPFs) and larger size (≥5 cm) of tumors.

## Additional Information

**How to cite this article**: Yan, L. *et al.* Clinicopathological significance of *c-KIT* mutation in gastrointestinal stromal tumors: a systematic review and meta-analysis. *Sci. Rep.* **5**, 13718; doi: 10.1038/srep13718 (2015).

## Figures and Tables

**Figure 1:**
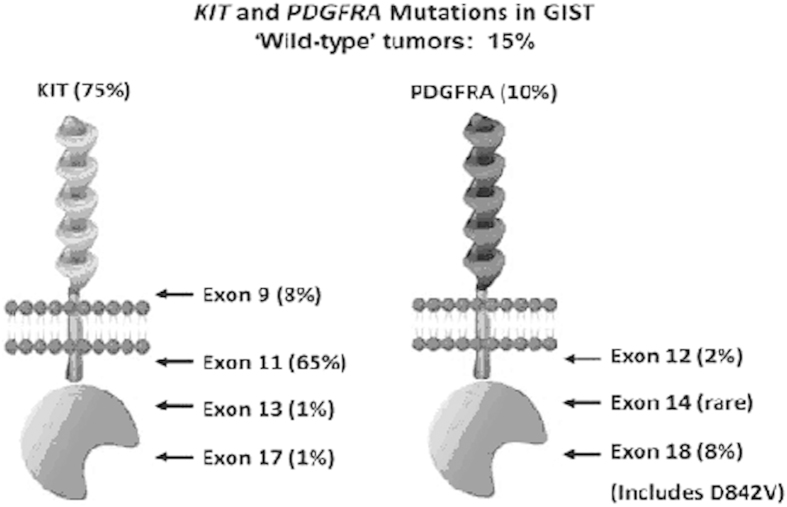
*KIT* and *PDGFRA* mutation in GIST.

**Table 1:** Main characteristics of included studies.

**Author**	**Year**	**Country**	Samplesize	Follow-up(Median)	**Treatment**
Ma *et al.*^31^	2014	China	68	91.3 mo	
Origone *et al.*^32^	2013	Italy	80		
Lv *et al.*^33^	2013	China	114	50 mo	Surgery
Kunstlinger *et al.*^34^	2013	Germany	1366		
Gao *et al.*^35^	2013	China	50	36 mo	Imatinib
Soreide *et al.*^36^	2012	Norway	38	8 year	Imatinib
Wozniak *et al.*^6^	2012	Belgium	427	3.8 year	Surgery
Kang *et al.*^37^	2012	Korea	370	43.3	Imatinib
Daniels *et al.*^38^	2011	Germany	87		
Garces-Albir *et al.*^28^	2012	Spain	36	64.8 mo	Surgery
Kontogianni-Katsarou *et al.*^39^	2008	Greece	30		
Tzen *et al.*^40^	2008	China	134	47 mo	
DeMatteo *et al.*^14^	2008	USA	127	5.2 year	Surgery
Imamura *et al.*^41^	2007	Japan	95	160 mo	
Debiec-Rychter *et al.*^42^	2006	Belgium	476	25.3 mo	Imatinib
Yeh *et al.*^43^	2006	China	64	16.1 mo	Imatinib
Cho *et al.*^44^	2006	Japan	56	56.3 mo	Imatinib
Martin *et al.*^45^	2005	Spain	162	42 mo	

Abbreviations: mo, month.
